# The case for an international patient-reported outcomes measurement information system (PROMIS^®^) initiative

**DOI:** 10.1186/1477-7525-11-210

**Published:** 2013-12-20

**Authors:** Jordi Alonso, Susan J Bartlett, Matthias Rose, Neil K Aaronson, John E Chaplin, Fabio Efficace, Alain Leplège, Aiping LU, David S Tulsky, Hein Raat, Ulrike Ravens-Sieberer, Dennis Revicki, Caroline B Terwee, Jose M Valderas, David Cella, Christopher B Forrest

**Affiliations:** 1 Health Services Research Unit, IMIM-InstitutHospital del Mar d’Investigacions Mèdiques, Barcelona, Spain; 2 CIBER Epidemiología y Salud Pública (CIBERESP), Spain; 3 McGill University/Royal Victoria Hospital, Montreal, Canada; 4 Department of Psychosomatic Medicine, Medical School CharitéUniversitätsmedizin, Berlin, Germany; 5 Department of Quantitative Health Sciences, University of Massachusetts, Massachusetts, MA, USA; 6 Department of Psychosocial Research, Division of Psychosocial Research & Epidemiology, The Netherlands Cancer Institute & Department of Clinical Psychology, The University of Amsterdam, Amsterdam, the Netherlands; 7 Institute of Clinical Sciences, Department of Pediatrics (Växthuset), The Queen Silvia Children's Hospital, Sahlgrenska Academy at the University of Gothenburg, Göteborg, Sweden; 8 Italian Group for Adult Hematologic Diseases (GIMEMA), Health Outcomes Research Unit, Rome, Italy; 9 Department of History and Philosophy of Science, Faculty of Life SciencesUniversity Paris, Paris, France; 10 School of Chinese Medicine, Hong Kong Basptist University, Kowloon Tong, Hong Kong, China; 11 Department of Physical Medicine and Rehabilitation, University of Michigan Medical School, Ann Arbor, MI, USA; 12 Erasmus MC, University Medical Center Rotterdam, Dept. of Public Health, Rotterdam, the Netherlands; 13 Department of Child and Adolescent Psychiatry, Psychotherapy, and Psychosomatics, University Medical Center Hamburg-Eppendorf, Hamburg, Germany; 14 Outcomes Research, Evidera, Bethesda, MD, USA; 15 Department of Epidemiology and Biostatistics and the EMGO Institute for Health and Care Research, VU University Medical Center, Amsterdam, the Netherlands; 16 Health Services and Policy Research Group, Department of Primary Care Health SciencesUniversity of Oxford, Oxford, UK; 17 Department of Medical Social Sciences, Feinberg School of Medicine, Northwestern University, Feinberg School of Medicine, Chicago, IL, USA; 18 University of Pennsylvania and Children's Hospital of Philadelphia, Philadelphia, PA, USA

**Keywords:** Patient-reported outcomes, Health-related quality of life research, Patients’ experiences, Questionnaires, Cross-cultural equivalence, Health information systems, Clinical decision making, Comparative effectiveness research, Patient empowerment, Cross-national comparisons

## Abstract

Patient-reported outcomes (PROs) play an increasingly important role in clinical practice and research. Modern psychometric methods such as item response theory (IRT) enable the creation of item banks that support fixed-length forms as well as computerized adaptive testing (CAT), often resulting in improved measurement precision and responsiveness. Here we describe and discuss the case for developing an international core set of PROs building from the US PROMIS^®^ network.

PROMIS is a U.S.-based cooperative group of research sites and centers of excellence convened to develop and standardize PRO measures across studies and settings. If extended to a global collaboration, PROMIS has the potential to transform PRO measurement by creating a shared, unifying terminology and metric for reporting of common symptoms and functional life domains. Extending a common set of standardized PRO measures to the international community offers great potential for improving patient-centered research, clinical trials reporting, population monitoring, and health care worldwide. Benefits of such standardization include the possibility of: international syntheses (such as meta-analyses) of research findings; international population monitoring and policy development; health services administrators and planners access to relevant information on the populations they serve; better assessment and monitoring of patients by providers; and improved shared decision making.

The goal of the current PROMIS International initiative is to ensure that item banks are translated and culturally adapted for use in adults and children in as many countries as possible. The process includes 3 key steps: translation/cultural adaptation, calibration, and validation. A universal translation, an approach focusing on commonalities, rather than differences across versions developed in regions or countries speaking the same language, is proposed to ensure conceptual equivalence for all items. International item calibration using nationally representative samples of adults and children within countries is essential to demonstrate that all items possess expected strong measurement properties. Finally, it is important to demonstrate that the PROMIS measures are valid, reliable and responsive to change when used in an international context.

IRT item banking will allow for tailoring within countries and facilitate growth and evolution of PROs through contributions from the international measurement community. A number of opportunities and challenges of international development of PROs item banks are discussed.

## Background

There is increasing recognition of the importance of integrating patients’ perspectives into clinical practice and research using tools that measure patients’ experiences of their health (i.e., patient-reported outcomes (PROs)). PROs first emerged in the late 1940’s [1] and since then their number and usage has increased dramatically. PROs can be used to assess symptoms, feelings, mood, behavior, health perceptions and attitudes, utilities, well-being, and health-related quality of life (HRQL) [2]. Well-known and internationally validated PRO measures, such as the SF-36 and EQ-5D, are used in clinical, economic and health services research, quality improvement, and clinical practice, as well as general population health assessment [3,4].

An exciting development in PROs has been the introduction of modern psychometric methods such as item response theory (IRT). IRT methods enable the creation of item banks that support multiple, interchangeable fixed-length forms and computerized adaptive testing (CAT). Advantages of IRT-based scales include lack of local dependence and interval scaling, resulting in precise measurement of the latent trait across the measurement continuum, which greatly improves responsiveness to change.

IRT-calibrated item banks are the methodological basis of the U.S. National Institutes of Health (NIH) Patient Reported Outcomes Measurement Information System (PROMIS^®^). Launched in 2004, PROMIS provides free access to over 60 item banks that have been calibrated and referenced to the US general population, with parallel evidence available in selected clinical populations [5]. One goal of PROMIS has been to standardize measurements in research and clinical practice, much like blood chemistry panels, thus facilitating comparability of data across studies and settings. Here, we make the case for extending this PRO standardization internationally, building on the highly successful U.S. PROMIS network initiative.

## Main text

### Elements of the US PROMIS network

**Figure 1:**
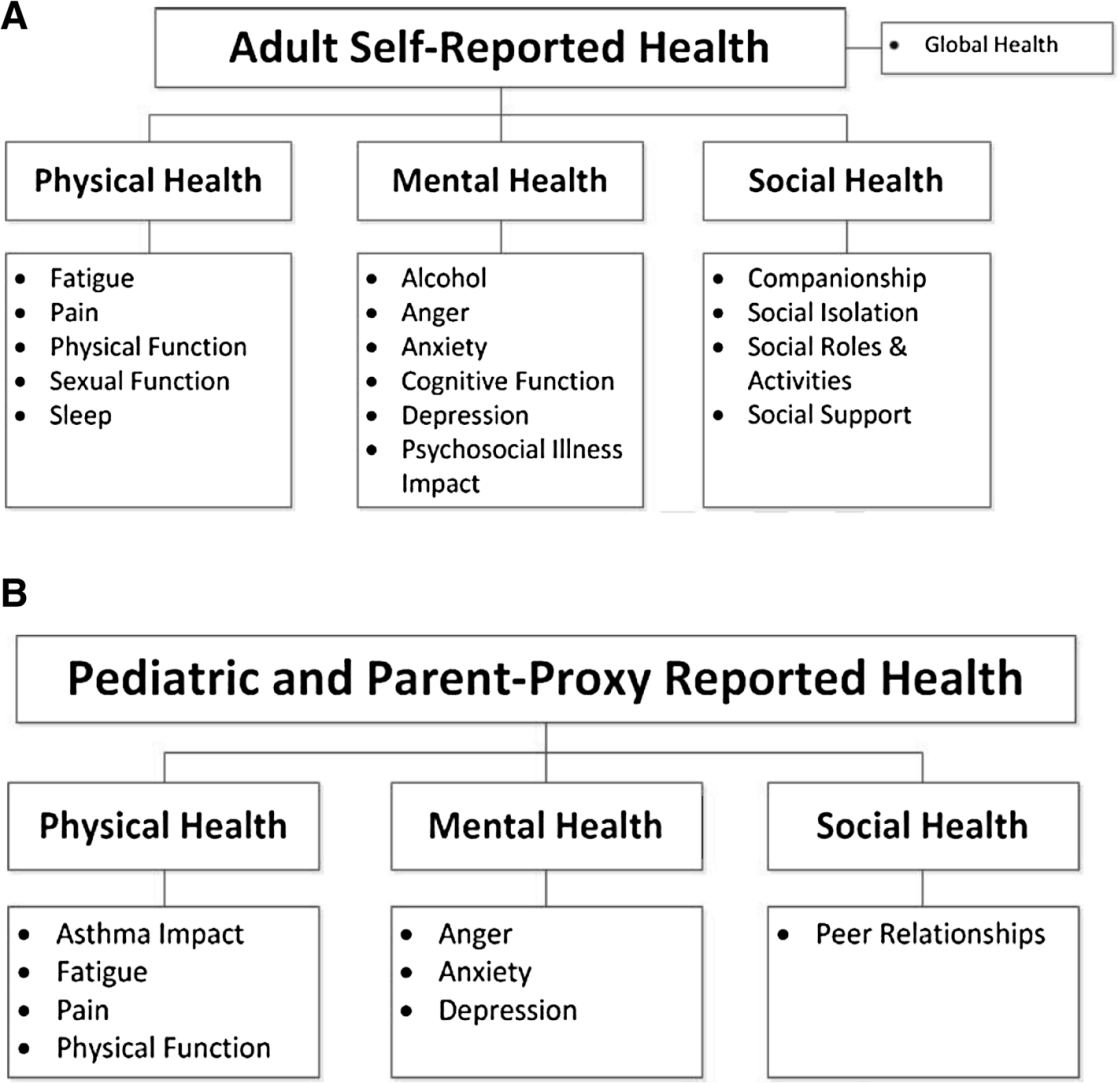
PROMIS domains available as of 2013: A. Adults; B. Pediatric and parent-proxy versions.

PROMIS is a cooperative group of research sites and centers that employ mixed-methods development processes (which include qualitative --e.g., focus groups and cognitive debriefing--, as well as quantitative methods --e.g., testing for differential item functioning using IRT theory) to create domain-specific measures of physical, mental and social health for use across diseases [5-8]. The domain-specific approach focuses on generic health constructs, such as pain, physical functioning, anxiety, and social isolation. Domain concepts are operationalized by large pools of item banks that are theoretically and empirically grounded in patient experiences and calibrated using IRT methods to produce item banks. Longitudinal validation studies are done to evaluate how sensitive the scales are to clinical change. Finally, domain specific fixed- and customized- short forms and dynamic CATs are generated to provide users with a variety of alternative administration options [5]. Current categories of PROMIS measures for adults and pediatrics are shown in Figure 1; several new PROMIS pediatric and adult measures are scheduled for release in 2014.

An important resource for domestic and international users of PROMIS is the Assessment Center, which is a web-based informatics platform that provides researchers with the tools needed to perform data collection of PROMIS and other PRO measures using the Internet [9]. PROs can be collected from patients via computers, tablets, or interactive voice response systems. Validated CAT algorithms are available for item banks [6]. The U.S. National Institutes of Health is providing financial support for Assessment Center until 2019 to maintain and distribute PROMIS instruments and scientific methods. After that date, these functions will be overseen and funded through a sustainable business model in collaboration with a newly formed entity called the PROMIS Health Organization, a non-profit organization that was developed by PROMIS investigators.

After less than a decade, the collaborative research of the US PROMIS network has seen rapid uptake of usage in clinical, translational, and pharmaceutical research. The initiative has advanced PRO measurement of key health domains, providing exhaustive and, in theory, universally applicable measures that can be reliably compared across settings and time. Several hundred researchers and an increasing number of clinicians have integrated PROMIS tools into their study protocols.

## Discussion

### Value to the international community

Extending a common set of standardized PRO measures to the international community offers great potential for improving patient-centered research, clinical trials reporting, population monitoring, and health care worldwide. Many pharmaceutical trials are now being done by recruiting patients from multiple countries. Cumulative worldwide PRO data can facilitate collaboration among a community of scientists to advance knowledge about patient experiences using a common set of concepts and measures. Comparability of results in multinational clinical trials and comparative effectiveness research increases the relevance and generalizability of results.

Adopting a common standard and metric will allow clinical researchers to directly compare patients’ evaluations of intervention effects from different samples and across countries, thereby increasing the relevance of results and enabling international syntheses (such as meta-analyses) of research findings. Results obtained through standardized PRO data collection can facilitate population monitoring and policy development. PROs can offer health services administrators and planners access to information that is of relevance and interest to their constituents and the populations they serve. Providers can use PROs to more comprehensively assess and monitor their patients’ health and treatment efficacy and safety, uncover hidden disability, the impact on health-related quality of life, and improve shared decision making. Thus, standardized PROs can become a key component of an international health information system supporting the provision of quality of health care as well as the advancement of scientific knowledge.

### Challenges and strategies

There are several potential challenges to widespread adoption of PROMIS as a standardized international PRO measurement system. A major consideration will be the costs associated with translation and cultural adaptation, calibration and validation, and training necessary for widespread use of global PROMIS measures. Identifying resources available at the national and international levels that can provide ongoing support for the PROMIS International initiative will be essential for the long-term sustainability of this effort. This likely will require coordination of investment from a range of national and international sources including governments, industry, foundations and health systems/payers. The PROMIS International initiative is growing in a phased approach, beginning with early adopter countries where there are scientific leaders and established research groups working on patient-reported outcomes. As PROMIS becomes an international standard, resources will be attracted for studies that include more resource-constrained countries, and PROMIS will grow with each of these international “use cases.”

Knowledge translation and educational efforts will be required to increase confidence in the added valued of using IRT-derived measures, while addressing longstanding concerns about the use of generic (versus disease-specific) instruments and the usefulness of PROs, in general. Training will be required to convince potential users that IRT-derived measures are indeed feasible to administer in a wide range of settings, easy to interpret, valid across countries and cultures and meaningful to users, and to accelerate the learning curve associated with the use of CAT.

While the PROMIS International initiative also brings opportunities for ongoing contributions from international researchers, the current model of governance will need to reflect shared leadership and clear governance processes and procedures, including the need to anticipate, identify and address potential conflicts of interest across borders.

## Conclusions

A robust and valid health outcomes measurement system based on patient input, yielding comparable health information across multiple languages and cultures, can contribute meaningfully to developed evidence-based interventions and health care cost containment. In the short term, the more modest goal of the current PROMIS International initiative is to ensure that item banks are translated and culturally adapted for use in adults and children in the most frequently spoken languages worldwide. Rigorous mixed-methods approaches are essential to ensure that items and instruments are equivalent across languages and cultures. Translation methods currently proposed are based on the systematic, multistage process of forward and backward translation and are based on the experience gained from previous international efforts to standardize PRO measures [10]. A “universal” approach (one translation per language) is proposed, where feasible, rather than a country- or dialect-specific version [11]. A focus on commonalities, rather than differences, ensures that all items are perceived similarly and use language appropriate for as wide a range of people as possible. One limitation of the universal translation approach used is that an item might not be expressed in the most popular/natural way of expressing a concept. To date, however evidence suggests that Spanish items behave as well as the original US English items.

A crucial component for cultural adaptation is international item calibration for each domain using nationally representative samples of adults and children within countries (or other significant subgroups). Calibrations help evaluate whether items demonstrate similar properties as the original PROMIS measures (i.e., unidimensionality, model fit, monotonicity, scalability, item fit, and item parameter invariance). Empirical evidence of psychometric performance (e.g., confirmation of factor structure, of links with “legacy” measures, and of disease score profiles) must demonstrate that validity, reliability and responsiveness to change measure up to international standards.

After all the translation standards have been followed, we will look at empirical data to test whether each item has exactly the same measurement properties as in the original version. Differential Item Functioning (DIF) analyses will be the most important analytical strategy. If statistically significant and metrically relevant DIF is identified, then a modification or a substitution of this item should be performed, with subsequent testing of equivalence with the original. When differences are found, IRT methods allow for a research strategy including: understanding the extent to which observed differences make an impact on final scores. In that case, the problematic item might be replaced by another item in the item bank that is equivalent and shows no cultural differences. Alternatively, both the original and the translated item can be reviewed for improvement as to make sure they are metrically equivalent.

An important advantage of IRT item banking is that it allows for tailoring within countries and facilitates growth and evolution of the measurement tool through contributions from the international measurement community. Also, flexibility is necessary to identify potentially problematic items and instruments that require modification. Thus, ongoing cultural evaluation of PROMIS can improve the metrics of existing measures while also advancing the development of items banks for new domains.

## Abbreviations

PROMIS: Patient-reported outcomes measurement information system; PRO: Patient-reported outcomes; IRT: Item response theory; CAT: Computerized adaptive testing; HRQL: Health-related quality of life; SF-36: Short form 36 items; EQ-5D: EuroQol 5 dimensions; NIH: National Institutes of Health.

## Competing interests

The authors declare that they have no competing interests.

## Authors’ contributions

JA, SB, MR and CBF conceived the paper and wrote the first draft. All coauthors reviewed the paper and have provided critical input to it. All authors read and approved the final manuscript.

## PROMIS International group

Neil K. Aaronson, Jordi Alonso, Susan J. Bartlett, Jakob Bjørner, John Brazier, John E. Chaplin, Helena Correia, Jocelyne M.R. Clench-Aas, Fabio Efficace, Pedro L. Ferreira, Francis Guillemin, Madelaine King, Alain Leplège, Aiping LU, David S. Tulsky, Hein Raat, Ulrika Ravens-Sieberer, Dennis Revicki, Matthias Rose, Caroline B. Terwee, Henrica de Vet, Jose M. Valderas, Christopher B. Forrest, and David Cella,
